# Functional Characterization of *BrF3'H*, Which Determines the Typical Flavonoid Profile of Purple Chinese Cabbage

**DOI:** 10.3389/fpls.2021.793589

**Published:** 2021-12-08

**Authors:** Sangkyu Park, Hyo Lee, Myung Ki Min, Jihee Ha, Jaeeun Song, Chan Ju Lim, Jinpyo Oh, Saet Buyl Lee, Jong-Yeol Lee, Beom-Gi Kim

**Affiliations:** ^1^Metabolic Engineering Division, National Institute of Agricultural Sciences, Rural Development Administration, JeonJu, South Korea; ^2^Institute of Biotechnology and Breeding, Asiaseed Inc., Icheon, South Korea

**Keywords:** flavonoid, anthocyanin, Chinese cabbage, *Brassica rapa*, F3'H, quercetin

## Abstract

Flavonols and anthocyanins are the two major classes of flavonoids in *Brassica rapa*. To elucidate the flavonoid biosynthetic pathway in Chinese cabbage (*B. rapa* L. subsp*. pekinensis*), we analyzed flavonoid contents in two varieties of Chinese cabbage with normal green (5546) and purple (8267) leaves. The 8267 variety accumulates significantly higher levels of quercetin, isorhamnetin, and cyanidin than the 5546 variety, indicating that 3′-dihydroxylated flavonoids are more prevalent in the purple than in the green variety. Gene expression analysis showed that the expression patterns of most phenylpropanoid pathway genes did not correspond to the flavonoid accumulation patterns in 5546 and 8267 varieties, except for *BrPAL1.2* while most early and late flavonoid biosynthetic genes are highly expressed in 8267 variety. In particular, the flavanone 3′-hydroxylase *BrF3*′*H* (*Bra009312*) is expressed almost exclusively in 8267. We isolated the coding sequences of *BrF3*′*H* from the two varieties and found that both sequences encode identical amino acid sequences and are highly conserved with *F3'H* genes from other species. An *in vitro* enzymatic assay demonstrated that the recombinant *Br*F3′H protein catalyzes the 3′-hydroxylation of a wide range of 4′-hydroxylated flavonoid substrates. Kinetic analysis showed that kaempferol is the most preferred substrate and dihydrokaempferol (DHK) is the poorest substrate for recombinant *Br*F3′H among those tested. Transient expression of *BrF3*′*H* in *Nicotiana benthamiana* followed by infiltration of naringenin and DHK as substrates resulted in eriodictyol and quercetin production in the infiltrated leaves, demonstrating the functionality of *Br*F3′H *in planta*. As the first functional characterization of *Br*F3′H, our study provides insight into the molecular mechanism underlying purple coloration in Chinese cabbage.

## Introduction

Chinese cabbage (*Brassica rapa* L. subsp. *pekinensis*) is an important vegetable mainly cultivated and consumed in East Asia. This vegetable is highly nutritious and beneficial to human health due to its abundant content of phytochemicals such as glucosinolates, phenolic acids, carotenoids, and flavonoids (Šamec et al., 2011; Park et al., 2019), which commonly exhibit antioxidant and anticancer effects (Nichenametla et al., 2006; Podsędek, 2007; Boots et al., 2008; Pandey and Rizvi, 2009). Chinese cabbage is the principal ingredient of kimchi, a traditional fermented food in Korea. Because global kimchi consumption has been rising in recent years, efforts to develop superior varieties of Chinese cabbage are increasingly gaining momentum.

Numerous sophisticated breeding methods have been used to generate new Chinese cabbage varieties with improved nutritional quality. For instance, a cross between green Chinese cabbage and turnip (*Brassica rapa* L. subsp. *rapa*) produced orange-colored Chinese cabbage, which has a deep orange inner head with significantly higher levels of carotenoids and phenolics than its Chinese cabbage parent (Watanabe et al., 2011). Moreover, purple Chinese cabbages were developed by crossing green Chinese cabbage with red bok choy (*Brassica rapa* L. subsp. *chinensis*; Jiang et al., 2013), purple flowering Chinese cabbage (He et al., 2016), and red cabbage (*Brassica oleracea* L. var. *capitata* f. *rubra*; Lee et al., 2018). Metabolic profiling of these hybrid purple Chinese cabbages showed increased phenolic acid and flavonol (kaempferol, quercetin, and isorhamnetin) contents in leaves. Additionally, anthocyanins, which are absent in green Chinese cabbage, were newly synthesized at remarkably high levels in purple Chinese cabbage. Notably, 3′-hydroxylated flavonoids, such as quercetin and cyanidin, are the primary forms of flavonols and anthocyanidins, respectively, in purple Chinese cabbage (Jiang et al., 2013; He et al., 2016; Lee et al., 2018).

**Figure 1:**
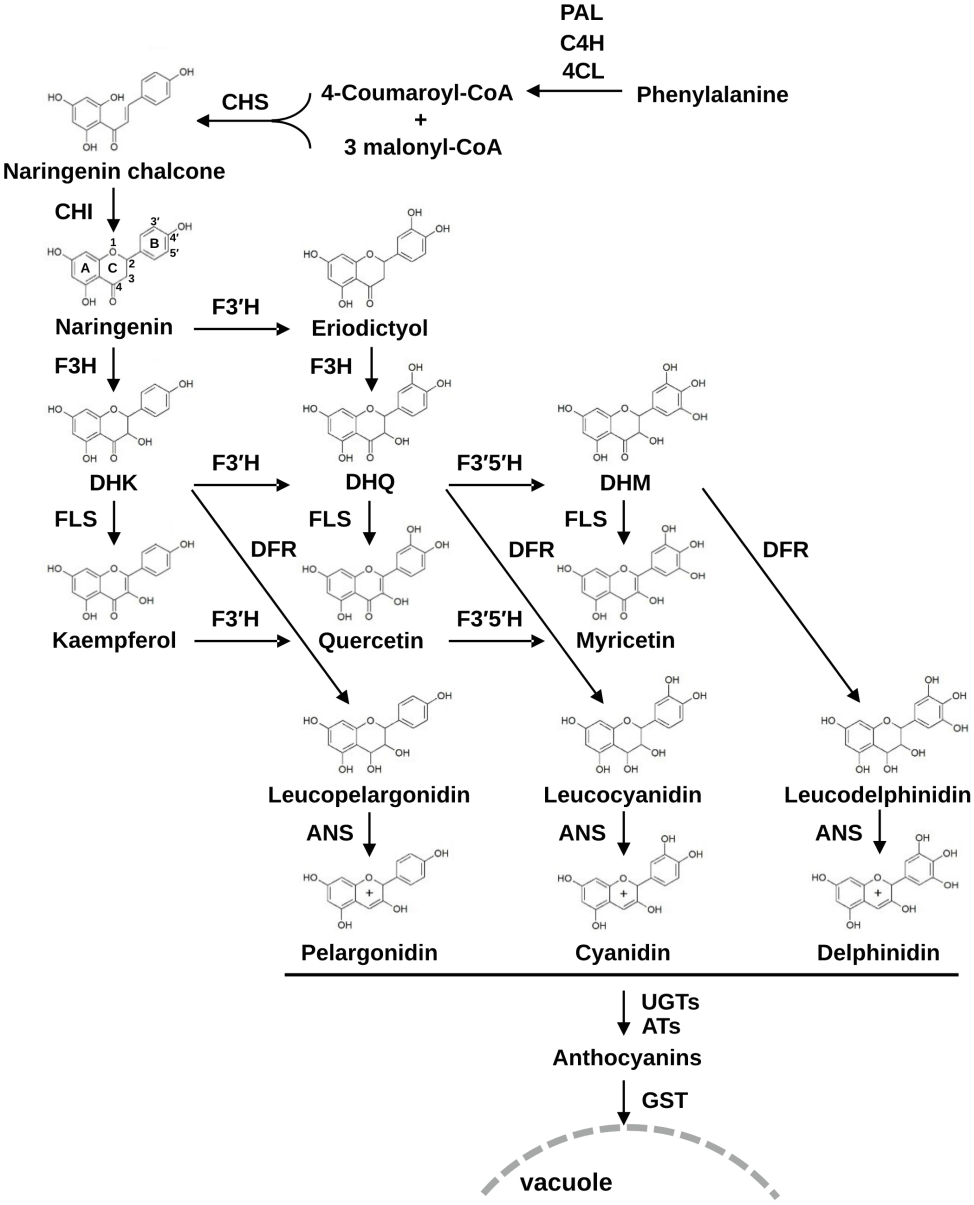
The flavonoid biosynthesis pathway in plants. PAL, phenylalanine ammonia-lyase; C4H, cinnamate 4-hydroxylase; 4CL, 4-coumaroyl:CoA-ligase; CHS, chalcone synthase; CHI, chalcone isomerase; F3H, flavanone 3*β*-hydroxylase; F3′H, flavonoid 3′-hydroxylase; F3′5′H, flavonoid 3′,5′-hydroxylase; DFR, dihydroflavonol 4-reductase; FLS, flavonol synthase; ANS, anthocyanidin synthase; UGTs, UDP-glucosyltransferase; ATs, acyltransferases; GSTs, glutathione *S*-transferases; DHK, dihydrokaempferol; DHQ, dihydroquercetin; DHM, dihydromyricetin.

The anthocyanin biosynthesis pathway is well characterized and generally conserved in plants (Forkmann, 1991; Xu et al., 2013; Li et al., 2016; Liu et al., 2016). The pathway is initiated from the conversion of phenylalanine to cinnamic acid by phenylalanine ammonia-lyase (PAL) and then consecutively catalyzed by cinnamate 4-hydroxylase (C4H), 4-coumaroyl:CoA-ligase (4CL), chalcone synthase (CHS), and chalcone isomerase (CHI) to form naringenin, a flavanone that acts as a universal substrate for flavonoid biosynthesis (Figure 1). Naringenin is catalyzed by flavanone 3-hydroxylase (F3H) to form dihydroflavonol dihydrokaempferol (DHK), which is further catalyzed by dihydroflavonol 4-reductase (DFR) and anthocyanidin synthase (ANS) to form the anthocyanidin pelargonidin. Anthocyanidins are subsequently modified by UDP-glucosyltransferases (UGTs) or acyltransferases (ATs) and transported to the vacuole by anthocyanin transporter glutathione *S*-transferases (GSTs). In another branch of the flavonoid biosynthesis pathway, DHK is converted to kaempferol by flavonol synthase (FLS). In this pathway, flavonoid 3′-hydroxylase (F3′H) or flavonoid 3′,5′-hydroxylase (F3′5′H) catalyzes hydroxylation of the B-ring with a broad range of substrate selectivity, which enables the production of eriodictyol from naringenin, dihydroquercetin (DHQ), or dihydromyricetin (DHM) from DHK, and quercetin or myricetin from kaempferol. As with DHK, DHQ, and DHM can be converted to anthocyanidin cyanidin and delphinidin, respectively, through DFR and ANS activities (Figure 1). Therefore, these B-ring hydroxylases are indispensable for generating major end-products of plant metabolism and are central to the variety of metabolic profiles in flavonoid production.

The *B. rapa* genome comprises 10 chromosomes (A01–A10) and has evolved from five chromosomes in Arabidopsis (*Arabidopsis thaliana*) through tandem gene duplication, whole-genome triplication (WGT), and fractionation of subgenomes (Wang et al., 2011; Cheng et al., 2012). Therefore, most *B. rapa* anthocyanin biosynthetic genes (ABGs) are found in multiple copies scattered on the 10 chromosomes (Guo et al., 2014). *B. rapa* ABGs have been identified through sequence homology- and synteny-based analysis of 51 orthologous Arabidopsis genes. A total of 86 mapped ABGs include phenylpropanoid metabolic pathway genes (*BrPAL*, *BrC4H*, and *Br4CL*), early flavonoid biosynthesis genes (EBGs; *BrCHS*, *BrCHI*, *BrF3H*, *BrFLS*, and *BrF3*′*H*), late flavonoid biosynthesis genes (LBGs; *BrDFR*, *BrANS*, *UGT*, *AT*, and *GST*), and several genes encoding regulatory proteins (such as *R2R3-MYB*, *bHLH*, and WD40-domain protein genes; He et al., 2020a). From these 86 genes, correlation analysis between gene expression and anthocyanin accumulation identified candidate genes responsible for anthocyanin biosynthesis in purple Chinese cabbage (He et al., 2020a,b). Twelve *BrDFR* paralogs and four *ANS* paralogs were extensively investigated at the transcript level in several other varieties of purple Chinese cabbage to identify genes required for anthocyanin production (Ahmed et al., 2014, 2015). However, the regulatory mechanism of flavonoid biosynthesis and the functional properties of most structural genes involved in these processes in Chinese cabbage still remain elusive.

According to previous reports (Jiang et al., 2013; He et al., 2016; Lee et al., 2018), the prevalence of 3′-hydroxylated flavonoids is characteristic of the purple Chinese cabbage varieties, indicating that anthocyanin production is typically accompanied by F3′H activity in purple Chinese cabbage. F3′H and F3′5′H are members of the cytochrome P450 enzyme superfamily. Cytochrome P450 enzymes (P450s) are localized mainly in the endoplasmic reticulum and catalyze an oxygenation reaction requiring NADPH, O_2_, and coupled activity of NADPH-cytochrome P450 reductase (CPR) that transfers electrons from NADPH to the P450 heme center (Huang et al., 2015). F3′H and F3′5′H enzymes are classified in P450 subfamilies CYP75B and CYP75A, respectively, with the exception of several Asteraceae F3′5′Hs belonging to CYP75B (Seitz et al., 2006). To date, these enzymes have been cloned and functionally analyzed in various monocot and dicot species (Menting and Stevenson, 1994; Shih et al., 2008; Falginella et al., 2010; Ishiguro et al., 2012; Sun et al., 2014; Lam et al., 2015; Park et al., 2016; Guo et al., 2019). In Brassicaceae species lacking *CYP75A* genes, however, only a few *CYP75B* genes have been cloned and functionally characterized; these include Arabidopsis *F3*′*H* (*AtF3*′*H*) and *B. napus F3*′*H* (*BnF3*′*H*). Unlike most structural genes of the flavonoid pathway, *BrF3*′*H* (*Bra009312*) exists as a single copy in the *B. rapa* genome, and its expression is correlated with anthocyanin accumulation in purple Chinese cabbage (He et al., 2020a,b). Thus, *Bra009312* is a possible candidate for functional *Br*F3′H.

In this study, we functionally characterized *BrF3′H* from purple Chinese cabbage commercially developed by crossing red flat cabbage (known as red tatsoi; *B. rapa* L. subsp. *narinosa*) and green Chinese cabbage. We analyzed the relationship between flavonoid profiles and the expression pattern of structural genes in the flavonoid pathway, along with the enzymatic properties of *Br*F3′H and its functionality *in planta*. Our data revealed the functional properties of *Br*F3′H, thereby providing an important basis for understanding flavonoid and anthocyanin biosynthesis in Chinese cabbage.

## Materials and Methods

### Plant Materials and Growth Conditions

A cross between red flat cabbage (red tatsoi; *B. rapa* L. subsp. *narinosa*) and two varieties of green Chinese cabbage (*B. rapa* L. subsp. *pekinensis*) followed by several backcrosses resulted in two different inbred purple heading Chinese cabbage lines. Crossing the two inbred lines gave rise to a purple heading F_1_ hybrid, ‘Jinhong Ssam,’ which was registered by the Asiaseed Company (Seoul, Korea). A further cross between ‘Jinhong Ssam’ and another elite line of green Chinese cabbage resulted in a second F_1_ hybrid; microspore cultures from this hybrid produced a doubled haploid (DH) line ‘8267’ with stable inheritance of purple heading. In this study, an inbred line with normal green traits (5546) and the DH line (8267) were used as the green and purple Chinese cabbage lines, respectively. Seeds of the two lines were surface sterilized, germinated, and grown on solid half-strength Murashige and Skoog medium under a 16-h-light/8-h-dark cycle at 26  ±  1°C for 10 days. The 10-day-old seedlings were transferred to soil and cultured for 65 days under the same conditions. Leaves of 10-, 45-, and 65-day-old plants were harvested and used to extract total RNA and flavonoids.

### Flavonoid Extraction and HPLC Analysis

Leaf flavonoid content of the 5546 and 8267 varieties was quantified as aglycone forms generated by acid hydrolysis with a slight modification of the previous method (Park et al., 2020). One hundred milligrams of ground leaf tissue was subjected to acid hydrolysis in 400 μl 50% methanol containing 2 N HCl at 94°C for 2 h. After centrifugation at 15,000 × *g* for 10 min at 4°C, the supernatant was transferred to a new tube. The remaining pellet was rinsed with 200 μl of the hydrolysis solution and pooled with the first extract. The extract was filtered through a 0.2-μm Teflon polytetrafluoroethylene (PTFE) hydrophilic syringe filter (Thermo Fisher Scientific, Waltham, MA, United States) and subjected to analysis performed on an LC-20A HPLC system (Shimadzu, Kyoto, Japan) equipped with an Inertsil-ODS3 C18 column (5 μm, 250 × 4.6 mm; GL Science, Eindhoven, Netherlands). The mobile phase consisted of 0.1% (v/v) formic acid in water (solution A) and 0.1% (v/v) formic acid in acetonitrile (solution B). The gradient profile was optimized as follows: 0–30 min, linear gradient of 5–55% (v/v) solution B; 30–45 min, linear gradient of 55–65% (v/v) solution B; 45–50 min, linear gradient of 65–100% (v/v) solution B at a flow rate of 1 ml·min^−1^. The temperature of the column was maintained at 40°C. A diode array detector was used for real-time monitoring of the chromatograms, and the spectra of the compounds were recorded between 210 and 800 nm. Flavanones and dihydroflavonols were analyzed at 288 nm, whereas flavonols and cyanidins were analyzed at 350 and 520 nm, respectively. The compounds were identified by comparing their retention times and UV spectra with those of flavonoid aglycone standards.

### Gene Expression Analysis

Total RNA was isolated from leaves of varieties 5546 and 8267 using the FavoPrep Plant Total RNA Mini Kit (Farvogen, Pingtung, Taiwan) according to the manufacturer’s instructions. First-strand cDNA was synthesized using anfiRivert cDNA Synthesis Platinum Master mix (GenDEPOT, Barker, TX, United States) and 2 μg of total RNA in a 20-μL reaction volume. The cDNA was diluted fourfold and used as a template for quantitative PCR (qPCR). The qPCR was performed in a 15-μL reaction containing AccuPower 2X GreenStar qPCR Master Mix (Bioneer, Daejun, Republic of Korea) and 0.4 μM of each primer (Table 1) using the Bio-Rad CFX96 detection system (Bio-Rad Laboratories, CA, USA) under the following conditions: 95°C for 5 min; 40 cycles at 95°C for 15 s and 55°C for 30 s. Each reaction was run in triplicate, and the amplification specificity was verified by melting curve analysis (55–95°C). Data were normalized to the expression levels of *B. rapa ACTIN7* (*BrACT7*; Table 1).

**Table 1:** Primers used for qPCR and cloning of *BrF3′H* coding sequence.

Target	Locus ID	Forward (5' to 3')	Reverse (5' to 3')
qPCR			
*BrPAL1.2*	Bra017210	TGAGCAAGTCTACACGTACGC	CGAAGTCATTGCATTCTTCTCGC
*BrPAL2.1*	Bra006985	AGTGTTAACCACTGGAGTCAACG	GAGACAAGGATCATCCACGTACG
*BrPAL2.3*	Bra003126	CTCATCTCGTAAGACCTCAGAAGC	CTGTCTCAGATTCTCCTCAAGATGC
*BrPAL3.2*	Bra030322	GTCTGATCTCCAGCCACAAGAC	AGATGTCTTAGATCATAGGCTTGGC
*BrC4H1*	Bra018311	GCTTGCTGGCTACGACATC	TCATTACCGTTCGCTTCCACG
*BrC4H2*	Bra021636	AGACCATCCGTAGAAGAATGGC	CCAGGCATTGATCAAGATTCTGC
*BrC4H4*	Bra022802	CTTGGTCAATGCATGGTGGCTA	GCAAGTACCTGAAGTCGTTACCG
*BrC4H5*	Bra022803	CATCAATGTCGCTGCTATTGAGAC	CCTGGTCCAAGAACCGTATCG
*Br4CL1*	Bra030429	GCTTCACACCGGAGATATCGG	CGTCGGTGATATCCTGATGGC
*Br4CL2.2*	Bra031263	AATGATCCGGAGGCTACTGC	AGCTGGAGCCACTTGATATCC
*Br4CL2.3*	Bra031265	AATGATCCGCAAGCTACTGC	AGCTGGAGCCACTTGATAGC
*Br4CL5.1*	Bra001819	TATCAAGTGGCTCCAGCTGAG	CACATATGCTACTGGAACCTCATC
*BrCHS1*	Bra008792	GGCTCTCTTCAGTGACGGC	GCACCGTCAGAGTCTGGTAAG
*BrCHS2*	Bra006224	AGTGAGTACGGAAACATGTCG	CAAGACAACAGTCTCTACGGTG
*BrCHS3*	Bra023441	ACATGTCTAGCGCCTGTGTC	CTGTGTAGGACAACTGTCTCC
*BrCHI1*	Bra007142	GCAGTTCTTGAATCGATCATTGG	GAGATCGTTAGCTTCCTCTTGATC
*BrF3H1*	Bra036828	ATCTTGGAGGAGCCAATTACG	CTAAGCGATGATTTGGTCTAGAGG
*BrFLS1*	Bra009358	CCAGTGTTCTTGGAGCCAC	TATTGAGCTTACGGTAACTGTAGTC
*BrF3′H*	Bra009312	TACGCCGGAGAAGCTGAACATG	TTAAGCCGACCCGAGTCCGTAAG
*BrDFR*	Bra027457	CAAAGTTCCGGGCAGTGATG	CTAAGCACAGATCTGCTGTGC
*BrANS1*	Bra013652	GTAAGCGCTCTGACCTTCATTCT	AGAATCTCCAACGTATCTCCAATATGC
*BrANS2*	Bra019350	CTTCATTCTACACAACATGGTTCCG	TACTAAGAATCTCCAACGTATCGCC
*BrUGT75C1*	Bra038445	CGTGGACTTGATCAGAGGAGATG	GAACACCGTTCTCTAAGCTCTCG
*BrUGT78D2*	Bra023594	ACTGACGAATAACCTCAAGTCGG	GCTACAGAACCAGTCCTCTGC
*BrTT8*	Bra037887	GTCAGAAGAGATAAGACTTGGTTCTCC	TGACATGAGAAGTGTTGATACTGTCTG
*BrMYB12.1*	Bra004456	CTTTAGGAGACTCTACGATAGCCAG	AACTCATCCGTCAACGCATC
*BtMYB12.2*	Bra000453	ATCTAGGAGACTCTGAAGAAGCC	ACTCATCCGTGAACGCATTATTG
*BrMYB113.1*	Bra004162	TAGATGAGAGCCAAGATCCAGC	GTTTCTCCATCCAACAGGCTC
*BrMYB113.2*	Bra039763	ATGCGGAGAAATATGAGCTCG	TTTCTGTCTCTGTAGATTCTGGAAC
*BrMYB113.3*	Bra001917	CAAAGCTGGGGAGAAGTATGAAC	GCCGTACCTTTTGGAACGAG
*BrACTIN7*	XM_009127097	GTGGATATCAGGAAGGATCTGTATGG	CAGACACTGTACTTCCTCTCAGG
Cloning			
*BrF3′H-CDS*		CACCATGACTAATCTTTACCTCACAATCCTTC	AGCCGACCCGAGTCCGTAAG

### Cloning and Yeast Expression of *BrF3*'*H*s

Full-length *BrF3′H* coding sequences lacking a stop codon were amplified by polymerase chain reaction (PCR) using first-strand cDNA from developing siliques of the 5546 variety and 65-day-old leaves of the 8267 variety, PrimeSTAR HS DNA polymerase (Takara, Otsu, Japan), and gene-specific primers (Table 1) under the following conditions: 98°C for 2 min; 33 cycles of 98°C for 10 s, 58°C for 10 s, and 72°C for 1 min 20 s; and a final extension at 72°C for 3 min. The PCR-amplified fragments were purified using a Gel Extraction Kit (Qiagen, Hilden, Germany), subcloned into pENTR/D-TOPO (Thermo Fisher Scientific), and verified by sequencing. As the open reading frame (ORF) sequences from 5546 and 8267 were identical, only the ORF from 8267 was cloned into the pYES-DEST52 yeast expression vector in frame with the V5 and 6 × His tags using Gateway LR™ clonase (Thermo Fisher Scientific) according to the manufacturer’s instructions. The resulting plasmid was transformed into the yeast strain WAT11 harboring genetically integrated Arabidopsis NADPH-cytochrome P450 reductase, *ATR1*, as a redox partner for the CYPs (Urban et al., 1997). Empty pYES-DEST52 was transformed as a negative control. Yeast transformation was performed as described previously (Gietz et al., 1992). Transformants selected on medium lacking uracil were grown in 50 ml of SGI medium (3.4 g·L^−1^ yeast nitrogen base, 5 g·L^−1^ Bacto Casamino acid, 40 mg·L^−1^ tryptophan, 150 mg·L^−1^ adenine hemisulfate, and 20 g·L^−1^ glucose) at 28°C for 24 h. The yeast cells were collected by centrifugation at 5000 × *g* for 5 min and resuspended with 50 ml of SLI medium (SGI medium supplemented with galactose instead of glucose). The resuspended yeast cells were incubated at 20°C for 72 h to induce recombinant *Br*F3′H protein expression. The induced cells were collected and subjected to microsome isolation.

### *In vitro* Assay of Recombinant *Br*F3'H Protein

The induced yeast cells were resuspended in 1.5 ml TEK buffer (50 mM Tris-Cl pH 7.5, 1 mM EDTA, and 100 mM KCl) and incubated at room temperature for 5 min. Cells were then collected by centrifugation (5,000 × *g*, 5 min, 4°C) and 400 μl TES buffer (50 mM Tris-Cl pH 7.5, 1 mM EDTA, 0.6 M sorbitol, 20 mM β-mercaptoethanol, and 1 mM phenylmethylsulfonyl fluoride) was added. The cells were disrupted by bead beating using glass beads under cold conditions and then centrifuged (5,000 × *g*, 5 min, 4°C) to collect the supernatant-containing microsomes. This process was repeated three times, and the supernatants were pooled. Subsequently, 150 mM NaCl and 0.1 g·ml^−1^ PEG 4000 were added to the supernatant before incubating on ice for 15 min to precipitate microsomes. The microsomal fractions were collected by centrifugation (10,000 × *g*, 10 min, 4°C) and resuspended in 500 μl TEG buffer (50 mM Tris-Cl pH 7.5, 1 mM EDTA, and 30% glycerol). The concentration of microsomal protein was determined by the Bradford method (Bradford, 1976) with bovine serum albumin as a standard. An *in vitro* assay was conducted in 100 μl reaction buffer containing 10 μg of the microsomal protein, 100 mM Tris-Cl (pH 7.5), substrate (one of the 4′-hydroxylated flavonoid substrates), and 1 mM NADPH (or without NADPH as a negative control) and incubated at 30°C for 20 min. The reaction was stopped by adding 500 μl of ethyl acetate and vortexing. After centrifugation, the upper ethyl acetate phase was transferred to a new tube and evaporated. The residue was dissolved in 100 μl of methanol and then subjected to HPLC analysis.

### Immunoblot Analysis

Galactose-induced or non-induced yeast cells were collected by centrifugation (5,000 × *g*, 5 min, 4°C) from 500 μl of yeast culture and resuspended with 100 μl of water and 100 μl of 0.2 M NaOH, followed by incubation at room temperature for 5 min. Cells were then collected and resuspended with 100 μl of 1 × yeast SDS sample buffer (60 mM Tris-Cl pH 6.8, 4% β-mercaptoethanol, 4% SDS, 0.01% bromophenol blue, and 5% glycerol) and boiled for 5 min. Yeast microsomal proteins were also prepared from galactose-induced cells harboring *BrF3′H* or the empty vector for immunoblot analysis. Ten microliters of total protein and 20 μg of microsomal protein were separated by 10% SDS-PAGE and electro-transferred onto a polyvinylidene fluoride membrane. After being blocked with skim milk in TBS-T buffer (50 mM Tris-Cl pH 7.4, 150 mM NaCl, 0.1% Tween 20, and 5% skim milk), the membrane was incubated with anti-His antibodies (Santa Cruz Biotechnology, Dallas, TX, United States) diluted 1/5000 at 4°C for 16 h. The membrane was washed with TBS-T buffer, and HRP-conjugated secondary antibodies (Thermo Fisher Scientific) diluted 1/5000 were then applied at 4°C for 2 h. Chemiluminescent signal was detected using West-Q Femto clean ECL solution (GenDEPOT) and the ImageQuant™ LAS 4000 system (Fujifilm, Tokyo, Japan).

### Agro-Infiltration of *Br*F3'H Into *Nicotiana benthamiana* Leaves

The full-length CDS of *BrF3′H* cloned into the pENTR/D-TOPO vector (Thermo Fisher Scientific) was transferred to the pB2GW7 binary vector (VIB-Ghent University, Ghent, Belgium) *via* a LR reaction. The resulting plasmid was transformed into *Agrobacterium tumefaciens* strain GV3101 by the freeze–thaw method (Holsters et al., 1978). The bacterial colony selected from solid LB medium containing rifampicin (50 μg·ml^−1^) and spectinomycin (50 μg·ml^−1^) was inoculated into 20 ml of liquid LB and incubated at 28°C for 16 h. This culture was centrifuged at 5000 × *g* for 10 min to pellet the cells; the pellet was then resuspended in 2 ml of infiltration medium (10 mM MES pH 5.6, 10 mM MgCl_2_, and 200 μM acetosyringone) and diluted to an OD_600_ of 0.5. After pre-incubation at room temperature for 2 h, the *Agrobacterium* suspension was infiltrated into the abaxial side of 4-week-old *N. benthamiana* leaves with a needleless 1-ml syringe. *Agrobacterium* harboring the empty pB2GW7 vector was simultaneously infiltrated into *N. benthamiana* leaves as a negative control. For substrate co-infiltration, infiltration medium supplemented with 100 μM naringenin or DHK substrate containing 0.1% DMSO was infiltrated into the same leaves 4 days after the initial agro-infiltration. The leaves were harvested 24 h later, and their metabolites were extracted using 50% methanol containing 2 N HCl for HPLC as described above.

### Chemical Standards

( ± )-Naringenin, ( ± )-eriodictyol, ( ± )-DHK, ( ± )-DHQ, kaempferol, quercetin, myricetin, and isorhamnetin were purchased from Sigma-Aldrich (St. Louis, MO, United States). Pelargonidin chloride, cyanidin chloride, and delphinidin chloride were purchased from Extrasynthese (Genay Cedex, France). The flavanones, dihydroflavonols, and flavonols were prepared as 100 mM stock solutions in DMSO, and the anthocyanidins were prepared as 100 mM stock solutions in 50% methanol containing 1.2 N HCl.

## Results

### Leaf Color Phenotypes and Flavonoid Content of Green and Purple Chinese Cabbage Varieties

**Figure 2:**
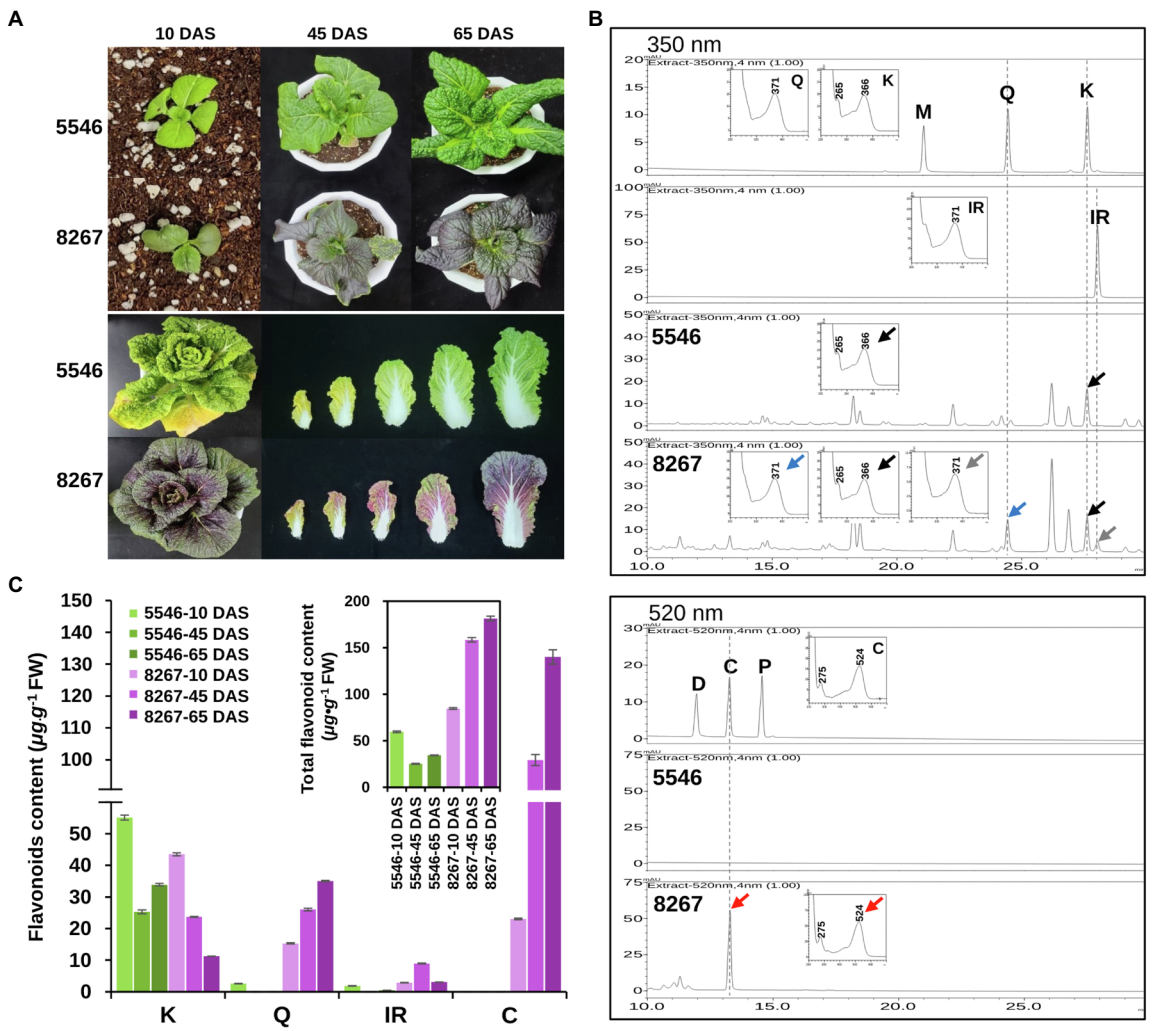
Comparison of phenotypes and flavonoid content of the 5546 and 8267 varieties. **(A)** Leaf color changes of the 5546 and 8267 varieties during growth. DAS, days after sowing. **(B)** HPLC analysis of flavonoid aglycones and anthocyanidins in leaves of 45-day-old plants of the two varieties. Flavonoid aglycones were separated by HPLC and detected at 350 nm for flavonols (M, myricetin; Q, quercetin; K, kaempferol; IR, isorhamnetin) and 520 nm for anthocyanidins (D, delphinidin; C, cyanidin; P, pelargonidin). Peaks were identified by comparing relative retention times and UV spectra of reference substances. Black, grey, blue, and red arrows indicate peaks in samples and their UV spectrums corresponding to K, IR, Q, and C, respectively. **(C)** Average content of the major classes of flavonoid and total flavonoids content in leaves of the two varieties at different growth stages are displayed in **(C)** and the inset of **(C)**, respectively. Error bars indicate ± SD from three replicates.

A comparison of the leaf color characteristics between varieties 5546 and 8267 during growth showed that, unlike 5546 seedlings, those of the 8267 variety had pale purple cotyledons at 10 days after sowing (DAS). The purple color darkened as the seedlings grew, and all true leaves of this variety displayed a strong purple color at 65 DAS (Figure 2A, upper panel). The full-grown head of 8267 also had a strong purple color, with the more mature outer leaves exhibiting a deeper color (Figure 2A, lower panel).

To compare the differences in flavonoid content of the two varieties, we harvested seedling leaves between 10 and 65 DAS. We extracted the leaves with acidic alcohol at 94°C to generate flavonoid aglycones and analyzed the extracts by HPLC (Figure 2B). The results showed that kaempferol is the major flavonoid in the 5546 leaves, while other classes of flavonoid aglycones were absent or hardly detected. In 8267 leaves, we detected aglycones of kaempferol, quercetin, isorhamnetin, and cyanidin (Figure 2B). Cyanidin levels were highest in 8267, sharply increasing at 45 DAS and reaching 130 μg·g^−1^ FW at 65 DAS (Figure 2C). These data indicate that cyanidin-derived anthocyanin is the main compound responsible for the purple trait observed in 8267. In terms of total flavonoids, the sum of flavonols and cyanidins in 8267 was higher than that in 5546 and increased as the 8267 variety grew, but this pattern was not observed in 5546 (inset of Figure 2C). These results suggest that flavonoid metabolic flux in 8267 is greater than that in 5546 and increases over time, whereas the flux in 5546 diminishes during growth.

### Expression Analysis of Flavonoid Biosynthetic Genes

**Figure 3:**
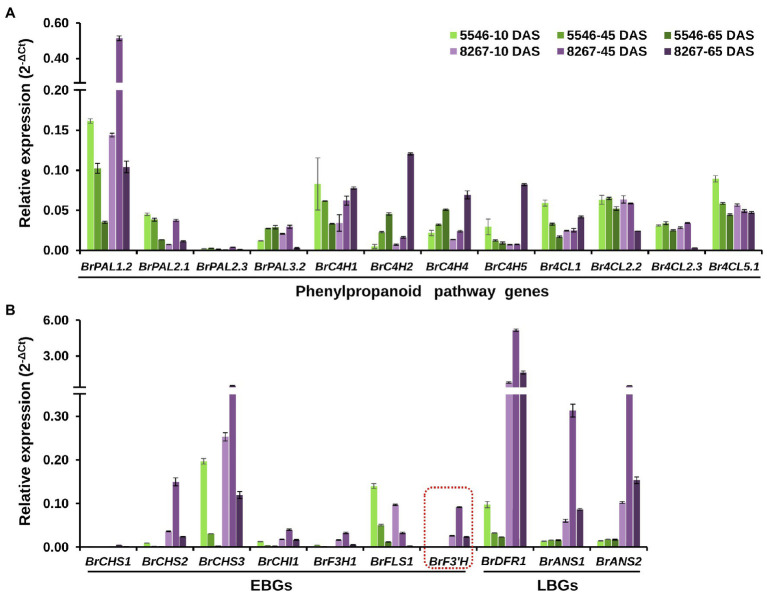
qPCR analysis of the early and late flavonoid biosynthetic genes. Relative expression levels of the **(A)** phenylpropanoid pathway genes (*BrPAL*, phenylalanine ammonia-lyase; *BrC4H*, cinnamate 4-hydroxylase; *Br4CL*, 4-coumaroyl:CoA-ligase), **(B)** early (*BrCHS*, chalcone synthase; *BrCHI1*, chalcone isomerase 1; *BrF3H1*, flavanone 3*β*-hydroxylase 1; *BrFLS1*, flavonol synthase 1; *BrF3′H*, flavonoid 3′-hydroxylase) and late (*BrDFR*, dihydroflavonol 4-reductase; *BrANS*, anthocyanidin synthase) flavonoid biosynthetic genes in leaves of the two varieties at different growth stages. Error bars indicate ± SD from three replicates. DAS, days after sowing.

We analyzed the expression levels of phenylpropanoid pathway genes and flavonoid biosynthetic genes in leaves of 5546 and 8267 by qPCR. Data were expressed as 2^-ΔCt^ to estimate the overall expression level of each gene tested (Figure 3). Among four *PAL* genes, the expression levels of *BrPAL1.2* were remarkably higher than those of the other paralogues in both varieties, and the expression in 8267 was predominantly higher than that in 5546 at 45 and 65 DAS, but this superior expression pattern in 8267 was not observed in the other *PAL* genes. Four *BrC4H*s expressed at similar or low levels in 8267 at 10 and 45 DAS compared to 5546, but their expression noticeably increased in 8267 at 65 DAS. In the case of *Br4CL*s, overall levels of their expression were comparable between 5546 and 8267, but *Br4CL2.2* and *Br4CL2.3* showed decreased expression levels in 8267 at 65 DAS (Figure 3A). These results indicate that only the expression pattern of *BrPAL1.2* was correlated with the changes in total flavonoid content in 5546 and 8267. Seven early flavonoid biosynthesis genes (EBGs), including *BrCHS2* and *BrCHS3*, were highly expressed in 8267. Notably, *BrF3*′*H* was expressed almost exclusively in 8267, possibly leading to the high degree of flavonoid biosynthesis and accumulation of 3′-hydroxylated metabolites such as quercetin and cyanidin derivatives in this line. The expression levels of EBGs decreased with time in 5546, whereas they peaked at 45 DAS and then fell in 8267, corresponding to the increase in quercetin and cyanidin. Interestingly, the expression levels of *BrFLS1* were similar in both varieties or slightly higher in 5546 at all growth stages, which is in agreement with the flavonol accumulation in the two varieties. We also examined the expression of three late biosynthesis genes (LBGs), *BrDFR1* and two paralogues of *BrANS*, and found that they were predominantly expressed in 8267. Like that of the EBGs, expression levels of the LBGs peaked at 45 DAS and then decreased, consistent with the drastic increase in cyanidin content observed in 8267 at the later growth stage (Figure 3B). Taken together, these results indicate that the expression patterns of *BrPAL1.2* and the flavonoid biosynthetic genes correlated with the differences in flavonoid and anthocyanin accumulation in the two varieties; particularly, the expression of *BrF3*′*H* corresponds to the abundant content of 3′-hydroxylated flavonoids in 8267 leaves.

### Sequence Analysis of *Br*F3'H

**Figure 4:**
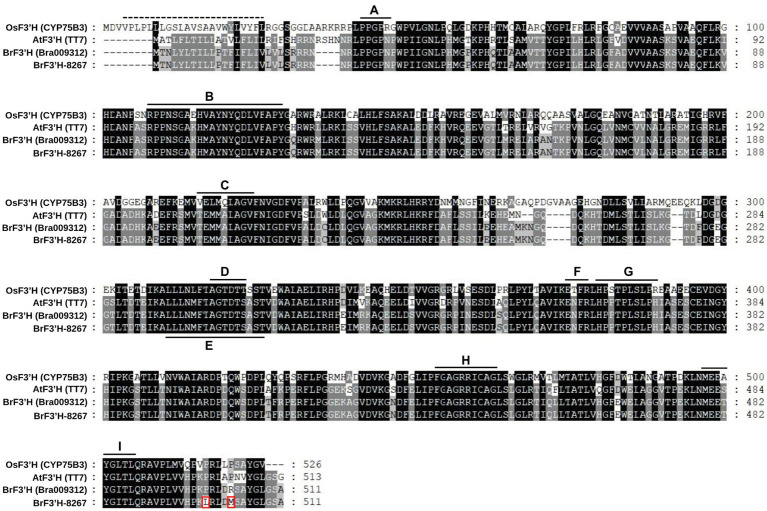
Amino acid sequence alignment of *Br*F3′Hs. The deduced amino acid sequence of *Br*F3′H from 8267 was compared with those of other known F3′Hs. Amino acid residues different from the reference sequence (*Bra009312*) are indicated with red rectangles. The dashed line located in the N-terminal region indicates the predicted membrane-spanning anchors, and solid lines indicate specific conserved regions of cytochrome P450 enzymes. A, hinge region; B, substrate recognition site 1 (SRS1); C, SRS2; D, oxygen binding pocket; E, SRS4; F, ExxR motif; G, SRS5; H, heme-biding domain; I, SRS6.

**Figure 5:**
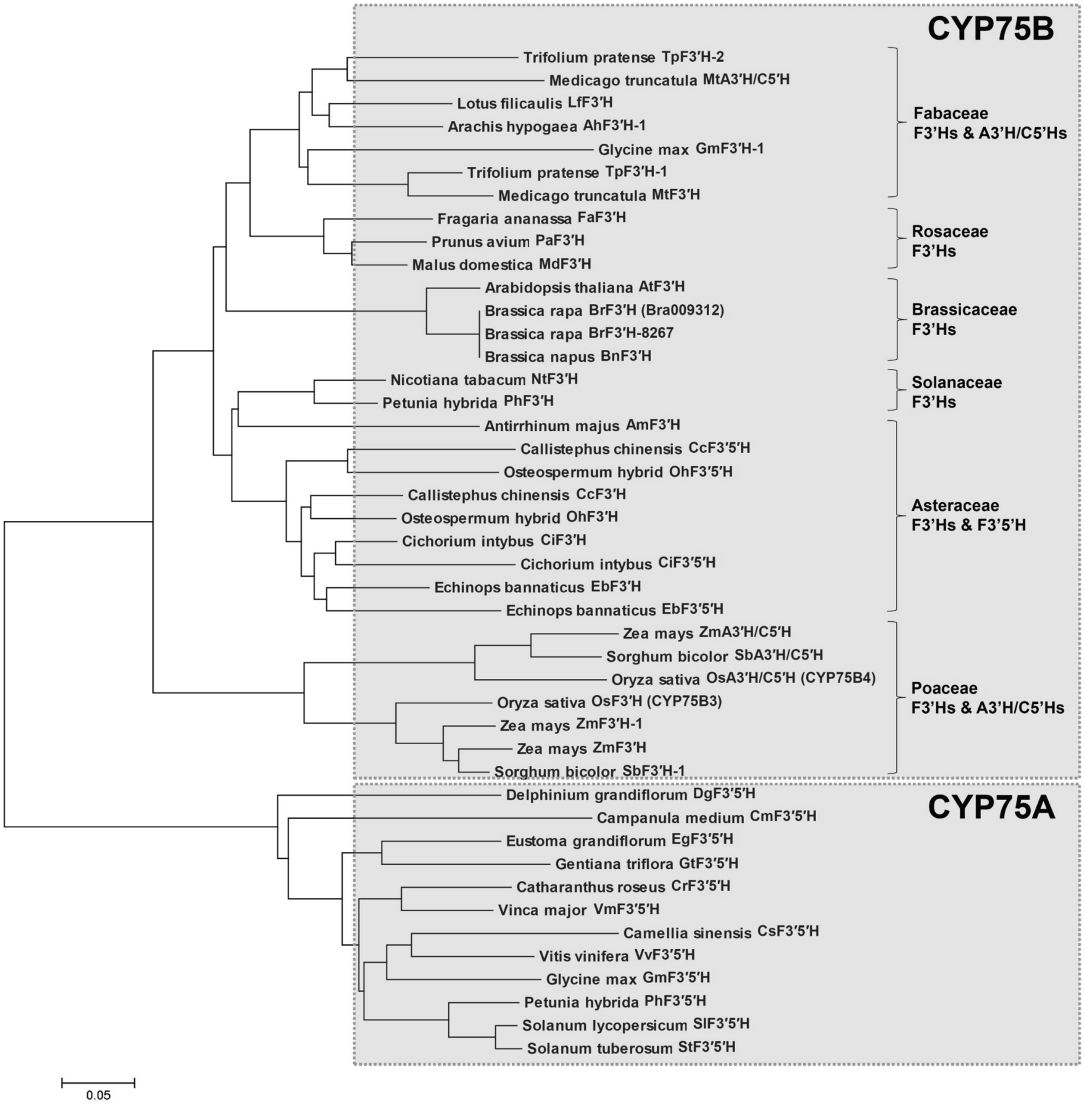
Phylogenetic analysis of flavonoid B-ring hydroxylases. The phylogenetic tree was generated based on amino acid sequence alignment of selected flavonoid B-ring hydroxylases and generated using the MEGA6 program (Scale bar, 0.05 amino acid substitution per site).

Because *BrF3*′*H* was exclusively expressed in 8267 seedlings, we cloned the coding sequences of *Bra009312* from siliques of 5546 and 65-day-old seedlings of 8267. PCR was conducted using a primer set designed according to the annotated sequence of *Bra009312* (Phytome v8.0/Brassica v1.2). The two sequences are identical and consist of 1,536 bp encoding 511 amino acids. Compared to the Bra003912 sequence, they show differences in 17 nucleotides (Supplementary Figure S1), but most are synonymous, resulting in only two amino acid differences, at the C terminus, when the sequences are translated (Figure 4). Deduced amino acid sequence alignment showed that *Br*F3′H from 8267 (*Br*F3′H-8267) shares 61.5 and 88.9% identity with rice (*Oryza sativa*) *Os*CYP75B3 and Arabidopsis *At*F3′H, respectively. The conserved regions of cytochrome P450s, such as the hinge region, oxygen binding pocket, ExxR motif, heme binding domain, and substrate recognition sites (SRSs), are identical in *Br*F3′H-8267 and *At*F3′H (Figure 4). We conducted phylogenetic analysis to further clarify the functional classification of *Br*F3′H-8267 within the family of flavonoid B-ring hydroxylases (Figure 5). *Br*F3′H-8267 was classified into the CYP75B family, to which general F3′Hs belong, and formed a distinct clade containing Brassicaceae F3′Hs, including *At*F3′H and *Bn*F3′H. These analyses imply that *Br*F3′H-8267 is an F3′H enzyme with only flavonoid 3′-hydroxylase activity, functionally similar to *At*F3′H and *Bn*F3′H (Schoenbohm et al., 2000; Xu et al., 2007).

### *In vitro* Enzymatic Assay of Recombinant *Br*F3'H

**Figure 6:**
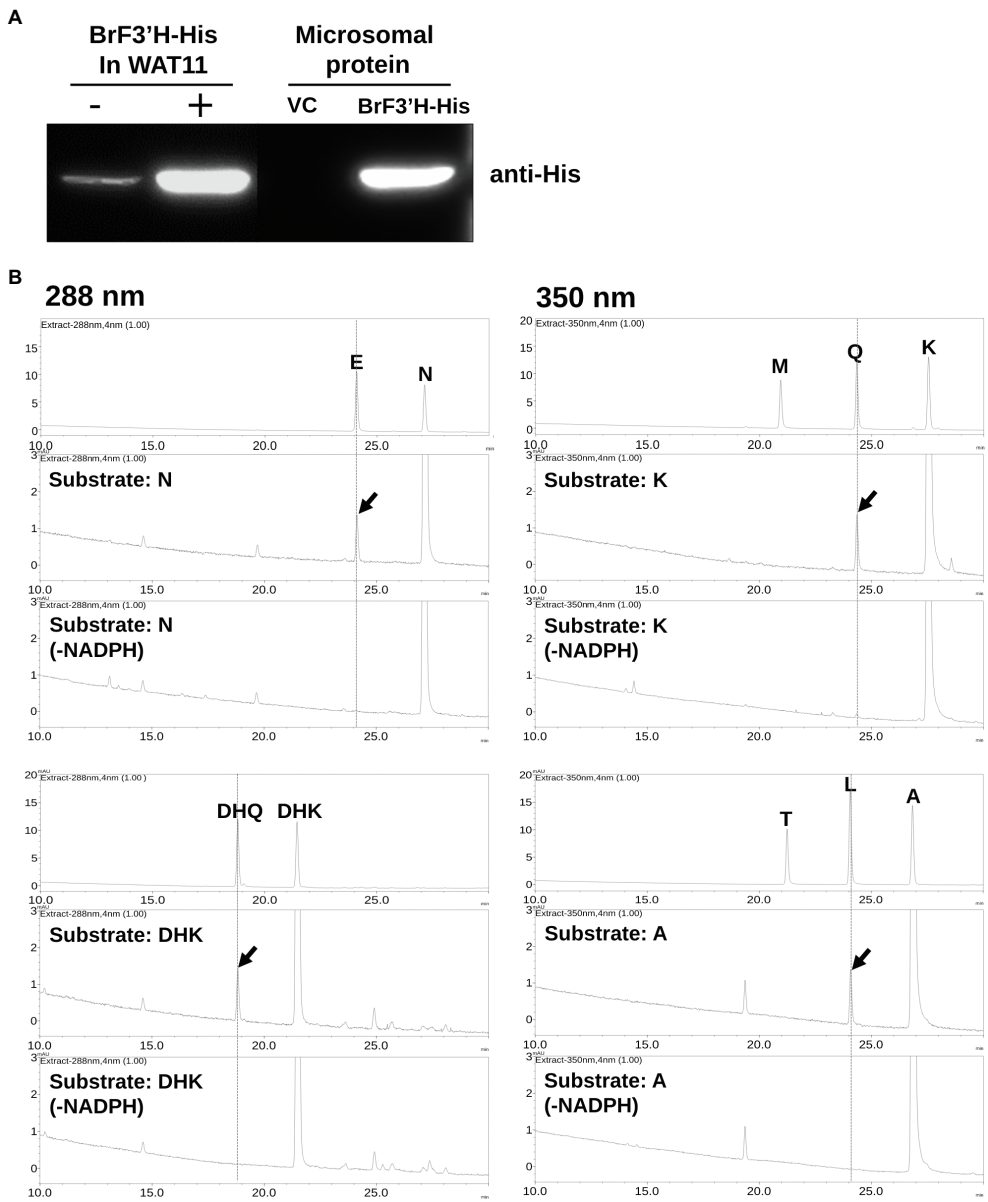
*In vitro* assay for recombinant *Br*F3′H-8267 protein. **(A)** Immunoblot analysis to verify the expression of recombinant His-tagged *Br*F3′H-8267 protein (*Br*F3′H-His) in yeast cells (left) and microsomal fraction (right). *Br*F3′H-His was detected using an anti-His antibody. – and +, non-induced and induced by IPTG; VC, vector control. **(B)** The *in vitro* reactions using recombinant *Br*F3′H protein were conducted along with the reactions without NADPH (-NADPH) as negative control, which were then analyzed using HPLC. Peaks were detected at 288 nm for dihydroflavonols (DHQ and DHK) and flavanones (E, eriodictyol; N, naringenin) and at 350 nm for flavonols (M, myricetin; Q, quercetin; K, kaempferol) and flavones (T, tricetin; L, luteolin; A, apigenin). The peaks were identified by comparing relative retention times and UV spectra to the standards, and peaks corresponding to 3′-hydroxylated flavonoids produced by recombinant *Br*F3′H-8267 are represented by red arrows.

To assay *Br*F3′H activity, we expressed a His-tagged *Br*F3′H in yeast strain WAT11. We isolated microsomal proteins, including the recombinant *Br*F3′H enzyme, and verified them by immunoblot analysis using an anti-His antibody (Figure 6A). We assayed the *in vitro* activity of the microsomal proteins with different classes of 4′-hydroxylated flavonoid substrates. The recombinant *Br*F3′H successfully catalyzed the 3′-hydroxylation of naringenin, DHK, kaempferol, and the flavone apigenin to produce eriodictyol, DHQ, quercetin, and luteolin, respectively (Figure 6B). These findings indicate that *Br*F3′H has broad flavonoid substrate specificity. However, we did not observe further hydroxylation to yield the 3′,5′-hydroxylated product from the 3′-hydroxylated products even with a prolonged reaction time. These results demonstrate that recombinant *Br*F3'H is a functional and canonical F3′H enzyme. To verify the catalytic efficiencies of *Br*F3′H for the substrates, we conducted a kinetic analysis with the 4′-hydroxylated flavonoid substrates except for apigenin. This yielded the lowest *K*_m_ value and the highest *V*_max_ value for kaempferol, thus making the catalytic efficiency for kaempferol the highest among the tested substrates (Table 2); meanwhile, intermediate and lowest levels of catalytic efficiency were observed for naringenin and DHK, which were numerically 3.4-fold and 17-fold lower than that for kaempferol, respectively. Thus, for the *in vitro Br*F3′H activity, the preferred substrate is kaempferol, while the least preferred is DHK.

**Table 2:** Kinetic parameters for 3'-hydroxylation activity of recombinant *Br*F3'H.

BrF3'H	Substrates		
	Nar	DHK	K
*K*_m_ ( μM)	1722 ± 152	3,604 ± 223	942 ± 65
*V*_max_ ( μM·min^**−**1^·mg^**−**1^)	3.52 ± 0.34	1.27 ± 0.11	6.27 ± 0.47
*V*_max_/Km (1·min^**−**1^·mg^**−**1^)	0.0020 ± 0.00122	0.0004 ± 0.00003	0.0067 ± 0.00056

### Transient Expression of *Br*F3'*H* in *Nicotiana benthamiana* Leaves

**Figure 7:**
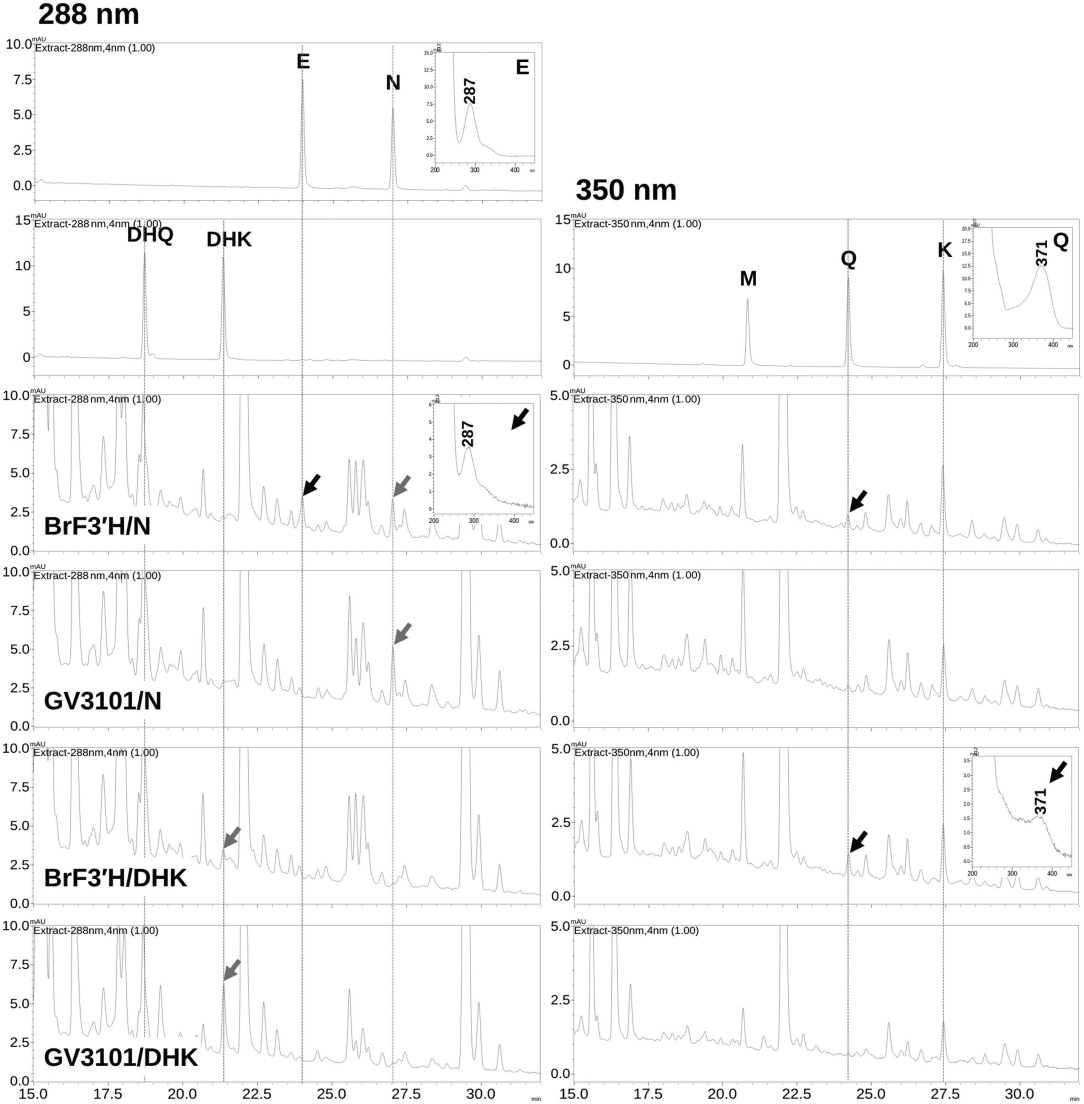
Production of 3′-hydroxylated flavonoids in *N. benthamiana* leaves by transient expression of *Br*F3′H-8267. *N. benthamiana* leaves were agro-infiltrated with GV3101 strain harboring *Br*F3′H-8267, and substrates (N, naringenin; DHK, dihydrokaempferol) were infiltrated into the same leaves 4 days after the initial agro-infiltration of *Br*F3′H and incubated for 24 h. GV3101/N and GV3101/DHK refer to leaves agro-infiltrated with the empty strain GV3101 as negative control and infiltrated with substrates (N or DHK) 4 days after the initial agro-infiltration. The leaves were extracted with acidic alcohol to generate flavonoid aglycones, and the extracts were analyzed using HPLC. Peaks were detected at 288 nm for flavanones (E, eriodictyol; N, naringenin) and dihydroflavonols (DHQ and DHK) and at 350 nm for flavonols (M, myricetin; Q, quercetin; K, kaempferol). The peaks were identified by comparing relative retention times and UV spectra to the standards, and peaks corresponding to the infiltrated substrates are indicated by black arrows and corresponding to 3′-hydroxylated flavonoids produced by the transient expression of *Br*F3′H-8267 are indicated by red arrows.

To verify *Br*F3′H-8267 functionality in the green Chinese cabbage, we attempted transient expression of BrF3'H-8267 by *Agrobacterium* (GV3101)-mediated infiltration but were unsuccessful. As an alternative, we agro-infiltrated *Br*F3'H-8267 into *N. benthamiana* leaves with posterior injection of naringenin or DHK as a substrate. The infiltrated tobacco leaves were extracted with acidic alcohol to generate flavonoid aglycones and analyzed by HPLC. The analysis showed that the peak areas corresponding to naringenin and DHK were reduced in the leaves expressing *Br*F3'H compared to the negative control (GV3101/N and GV3101/DHK), indicating that the substrates were consumed by *Br*F3'H successfully expressed in the tobacco leaves. In the leaves infiltrated with *Br*F3'H and naringenin, a peak corresponding to eriodictyol was newly generated, and the peak area corresponding to quercetin remarkably increased compared to the negative control (Figure 7), indicating that naringenin was converted to eriodictyol by transient expression of *Br*F3′H and further converted to quercetin by endogenous activities of F3H and FLS. It is also possible that endogenously accumulated kaempferol was directly converted to quercetin by *Br*F3′H. In the leaves infiltrated with *Br*F3′H and DHK, the peak area corresponding to quercetin increased significantly compared to the negative control, suggesting that the quercetin was produced from the DHQ generated from the infiltrated DHK or directly converted from endogenous kaempferol by *Br*F3′H activity. Taken together, these results demonstrate that *Br*F3′H is a functional enzyme for the 3′-hydroxylation of flavonoids *in planta*.

## Discussion

The previously reported hybrid varieties of purple Chinese cabbage commonly accumulate high levels of cyanidins and substantial levels of the flavonols quercetin, kaempferol, and isorhamnetin (Jiang et al., 2013; He et al., 2016; Lee et al., 2018). Similarly, the purple 8267 variety showed a flavonoid profile consisting of relatively high levels of both cyanidins and flavonols; by contrast, the green 5546 variety mainly accumulated kaempferol and lacked cyanidins (Figures 2B,C). These findings suggest that FLS activity plays an important role in the pathway for both varieties, whereas the combined activities of anthocyanin biosynthetic enzymes and F3′H are unique to the purple 8267 variety. Previous studies have indicated that the total contents of flavonoids, including flavonols and anthocyanins, are significantly higher in purple Chinese cabbage varieties than in green Chinese cabbages. We observed a similar pattern in our study, indicating that the metabolic flux for flavonoid biosynthesis is enhanced in purple Chinese cabbage. Collectively, these data suggest that anthocyanin accumulation in purple Chinese cabbages is associated with activation of the flavonoid pathway as well as with F3′H activity.

Gene expression analysis revealed a relationship between gene expression and anthocyanin accumulation in the 8267 purple Chinese cabbage variety. Recently, researchers identified key ABGs whose expression is tightly correlated with anthocyanin accumulation in purple Chinese cabbage that inherited the purple trait from purple flowering Chinese cabbage (He et al., 2020a). Their work demonstrated that except for *BrFLS1*, the expression of EBGs such as *BrCHS2*, *BrCHS3*, *BrCHS4*, *BrCHI1*, *BrF3H1*, *BrF3H3*, and *BrF3′H*, as well as LBGs such as *BrDFR1*, *BrANS1*, and *BrANS2*, was highly correlated with anthocyanin accumulation. Another study demonstrated that purple Chinese cabbage inherited *BrMYB2*, which acts as a principal regulator of anthocyanin production, from the purple flowering Chinese cabbage (He et al., 2020c). Similarly, our gene expression analysis showed that most EBGs and LBGs were highly expressed in 8267, except for *BrFLS1*. Therefore, it is conceivable that a key regulator such as *BrMYB2* was introduced into 8267 from the red tatsoi, which may explain why anthocyanin accumulation is accompanied by F3′H activity in this variety. Furthermore, phenolic acids such as caffeic acid, *p*-coumaric acid, ferulic acid, and sinapic acid are reported to accumulate to high levels in purple cabbages (Jiang et al., 2013; Lee et al., 2018). However, He et al. showed that most phenylpropanoid metabolic pathway genes, other than *BrPAL3.1* and *BrC4H4*, were down-regulated in the purple variety despite anthocyanin accumulation. Our study showed that, except for *BrPAL1.2*, the expression patterns of most phenylpropanoid pathway genes were not correlated with the changes in total flavonoid contents in 5546 and 8267 varieties thus it can be suggested that the enhanced flavonoid metabolic flux in the purple varieties was possibly due to the activation of EBGs such as *BrCHS*, *BrCHI*, *BrF3H*, and *BrF3′H* with the contribution of the phenylpropanoid pathway gene such as *BrPAL*.

In this study, we found that *BrF3′H* was exclusively expressed in 8267 seedlings; thus, we cloned the *BrF3’H* coding sequences from the siliques of 5546 and from 65-day-old seedlings of 8267, respectively. Both sequences were identical, confirming that the absence of 3′-hydroxylated flavonoids in 5546 was not due to mutations in *BrF3′H*. Phylogenetic analysis indicated that *Br*F3′H-8267 belongs to the CYP75B subfamily and forms a clade with *At*F3′H and *Bn*F3′H that exhibit only F3′H activity (Schoenbohm et al., 2000; Xu et al., 2007). To date, no other B-ring 5′-hydroxylases belonging to the CYP75A subfamily have been found in Brassicaceae. Nevertheless, 5′-hydroxylated or modified anthocyanidins such as delphinine, petunidin, and malvidin occur at trace levels in purple flowering Chinese cabbage and red cabbage (Lin et al., 2008; He et al., 2016), suggesting the possible existence of F3′5′H in Brassicaceae plants.

We analyzed the enzymatic properties of the recombinant *Br*F3′H-8267 protein through *in vitro* assays. Recombinant *Br*F3′H-8267 catalyzed the 3′-hydroxylation of naringenin, DHK, kaempferol, and apigenin. However, their 3′,5′-hydroxylated products were not found even in a prolonged reaction, demonstrating that recombinant *Br*F3′H-8267 is a functional enzyme exhibiting only F3′H activity for a broad range of flavonoid substrates. Subsequently, we conducted kinetic analysis of recombinant *Br*F3′H-8267 on the three main substrates, which showed that kaempferol is the favored substrate and DHK is the poorest substrate. Numerically, the protein’s catalytic efficiency for kaempferol was approximately 17 times higher than that for DHK. Given that *Br*F3′H prefers kaempferol over DHK, DHK should be preferentially converted to kaempferol by *Br*FLS. The kaempferol would then be converted to quercetin by *Br*F3′H. Eventually, quercetin is accumulated predominantly in 8267. However, considering the flavonoid composition of 8267, in which cyanidin is the major metabolite (Figure 2C), the substrate preferences are somewhat contradictory. To understand this contradiction, a metabolon-based interpretation is required. The metabolon is an enzymatic complex of sequentially arranged flavonoid biosynthetic enzymes, which facilitate direct substrate transfer or channeling of active sites (Winkel-Shirley, 1999). F3′Hs are ER membrane-anchored P450s and are thought to be a scaffold of the metabolon, thus playing a pivotal role in sequential enzymatic reactions. Shih et al. suggested that the metabolon in rice consists of CHS, F3H, F3′H, DFR, and ANS without FLS (Shih et al., 2008), which comprise a direct pathway to anthocyanin biosynthesis. When considering the metabolon in the flavonoid metabolism of 8267, it may be inferred that an increase in *Br*F3′H causes an increase in the number of metabolons, which promotes cyanidin biosynthesis directly from naringenin without the release of intermediates. Eventually, cyanidin would be the major metabolite in this variety.

The functionality of *Br*F3′H-8267 *in planta* was confirmed by agro-infiltration. Although our HPLC system did not detect changes in DHQ content, eriodictyol and quercetin were found to be produced by transiently expressed *Br*F3′H-8267 using infiltrated naringenin and DHK or endogenous kaempferol as substrates, indicating that BrF3′H is a functional enzyme *in planta*.

Taken together, we first identified enzymatic properties of *Br*F3′H and its functionality *in planta*, and the enhanced expression of *BrF3′H* is a critical point for the typical flavonoid profile in leaves of 826. Since *Br*F3′H is a key enzyme that determines the flavonoid metabolic profile characteristics in purple Chinese cabbage, the functionality of *Br*F3′H should be considered an important factor in the development of nutritionally improved varieties of Chinese cabbage. Further studies related to the metabolon in the flavonoid biosynthesis pathway and properties of other structural genes such as *BrF3H*, *BrFLS*, and *BrDFR* will greatly improve our understanding of flavonoid and anthocyanin biosynthesis in Chinese cabbage.

## Data Availability Statement

The datasets presented in this study can be found in online repositories. The names of the repository/repositories and accession number(s) can be found in the article/Supplementary Material.

## Author Contributions

SP performed gene cloning, gene expression analysis, HPLC analysis, and enzyme assay and wrote the manuscript. HL performed HPLC analysis, and enzyme assay. MM, JH, JS, CL, JO, SL, and J-YL contributed to the Chinese cabbage cultivation and the data curation and interpretation. B-GK supervised this study and revised the final version of the manuscript. All authors contributed to the article and approved the submitted version.

## Funding

This work was supported by the New Breeding Technologies Development Program (PJ014773) of the Rural Development Administration, Republic of Korea.

## Conflict of Interest

JO and CL were employed by company Asiaseed Inc.

The remaining authors declare that the research was conducted in the absence of any commercial or financial relationships that could be construed as a potential conflict of interest.

## Publisher’s Note

All claims expressed in this article are solely those of the authors and do not necessarily represent those of their affiliated organizations, or those of the publisher, the editors and the reviewers. Any product that may be evaluated in this article, or claim that may be made by its manufacturer, is not guaranteed or endorsed by the publisher.
