# Supplementary figures and images for: Functional Characterization of BrF3'H, Which Determines the Typical Flavonoid Profile of Purple Chinese Cabbage
Source: Front Plant Sci. 2021 Dec 8;12:793589. doi: 10.3389/fpls.2021.793589 (PMC8693655; doi:10.3389/fpls.2021.793589)

Supplementary Material


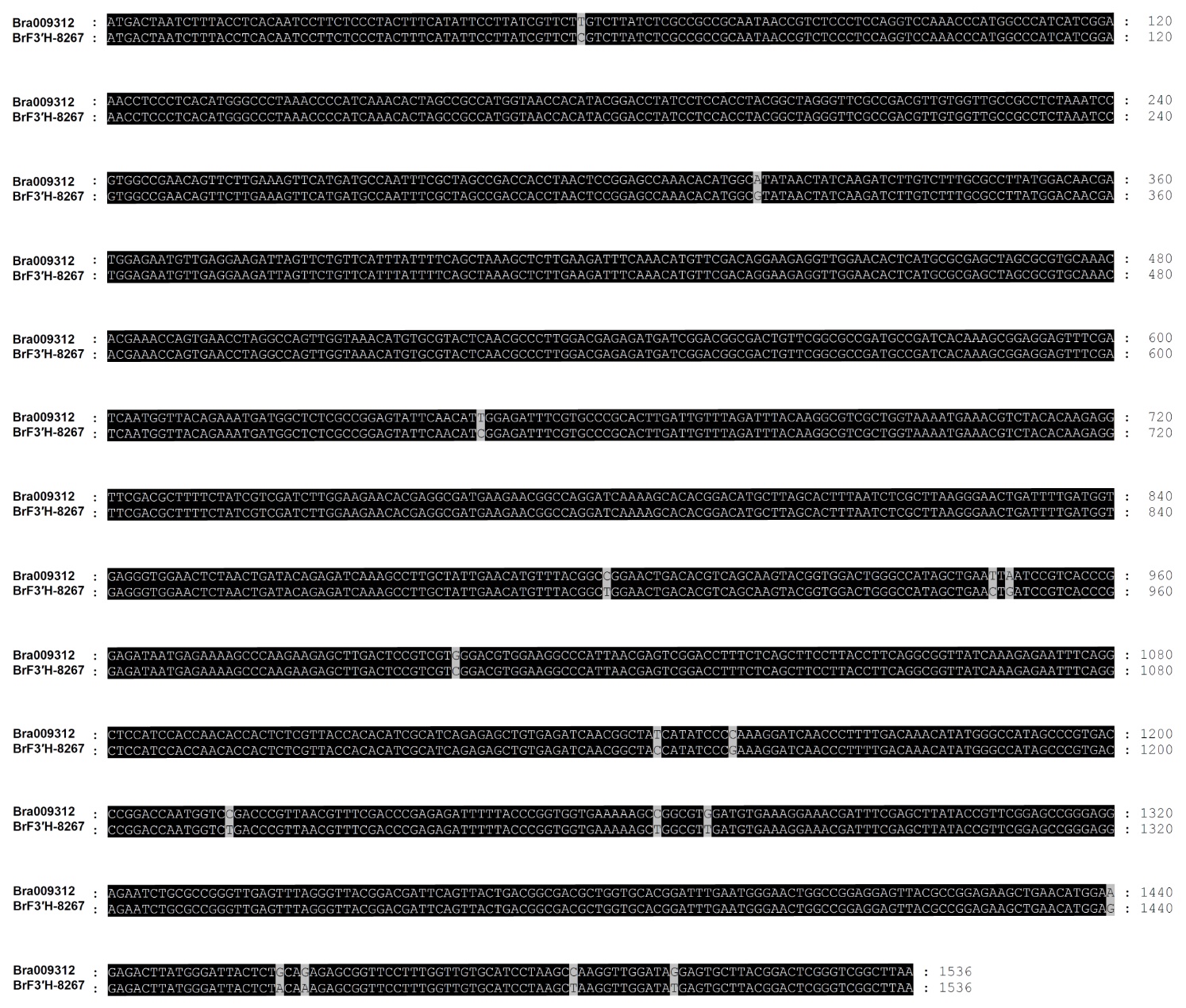


**Supplementary Figure 1.** Comparison of coding sequences of *Bra009312* and *BrF3′H-8267*

Supplement: Supplementary file 1 [file Data_Sheet_1.DOCX]
